# Quality protein intake is inversely related with abdominal fat

**DOI:** 10.1186/1743-7075-9-5

**Published:** 2012-01-27

**Authors:** Jeremy P Loenneke, Jacob M Wilson, Anssi H Manninen, Mandy E Wray, Jeremy T Barnes, Thomas J Pujol

**Affiliations:** 1 Department of Health and Exercise Science, University of Oklahoma, Norman, Oklahoma, USA; 2 Department of Health Sciences and Human Performance. University of Tampa, Tampa, Florida, USA; 3 Manninen Nutraceuticals Oy, Oulu, Finland; 4 Department of Health, Human Performance, and Recreation. Southeast Missouri State University, Cape Girardeau, Missouri, USA

## Abstract

Dietary protein intake and specifically the quality of the protein in the diet has become an area of recent interest. This study determined the relationship between the amount of quality protein, carbohydrate, and dietary fat consumed and the amount of times the ~10 g essential amino acid (EAA) threshold was reached at a meal, with percent central abdominal fat (CAF). Quality protein was defined as the ratio of EAA to total dietary protein. Quality protein consumed in a 24-hour period and the amount of times reaching the EAA threshold per day was inversely related to percent CAF, but not for carbohydrate or dietary fat. In conclusion, moderate to strong correlations between variables indicate that quality and distribution of protein may play an important role in regulating CAF, which is a strong independent marker for disease and mortality.

## Introduction

Dietary protein intake and specifically the quality of the protein in the diet has become an area of recent interest, particularly when combined with resistance training (for a thorough review the reader is directed to ref. [1]). Quality of protein is defined as the ratio of essential amino acids (EAA) to dietary protein in grams. The dietary reference intake (DRI) includes no specific recommendation regarding the type of dietary protein consumed or distribution of that dietary protein throughout the day. Approximately 10 g of EAA, at a meal, maximally stimulates muscle protein synthesis (MPS) [2]. EAA intake beyond this level does not appear to result in an additional anabolic response [3].

Studies have demonstrated that the consumption of dietary protein above the DRI has been associated with favorable changes in body composition [4]. Proposed mechanisms include the maintenance or accretion of lean mass and/or increased thermogenesis and satiety [5]. A 5-year prospective study found that protein intake was inversely related to changes in waist circumference [6]. Waist circumference is a surrogate marker for abdominal obesity, and this type of obesity is associated with significant risks of developing type 2 diabetes, coronary artery disease, stroke, and a higher risk of mortality, even after adjustments for general obesity [6]. However, the quality of the protein source consumed and the distribution of that protein throughout the day with respect to central abdominal fat (CAF) have not been investigated in free living conditions.

We sought to determine the relationship between the amount of quality protein consumed in 24-hours and the amount of times the ~10 g EAA threshold was reached at a meal, with respect to percent CAF. This is a secondary analysis using a data set from a previously reported paper on quality protein, overall body composition (lean mass and total body fat), and bone health [7].

## Methods

Twenty-seven healthy males (n = 12) and females (n = 15) (22 ± 3 yrs.; 169.68 ± 8.20 cm; 71.7 ± 13.9 kg; 34.2 ± 10.4% CAF) participated in this cross sectional study which was approved by the university's institutional review board. Subjects self-reported the amount of minutes (min) each week they participated in aerobic (174 ± 244 min) and resistance (93 ± 106 min) exercise. EAA intake was determined from a 3-day food record, and amino acid profiling for each food was determined using a computer program (Nutrition Data via USDA National Nutrient Database for Standard Reference, Release 22). The daily food records were averaged across the 3-days to give an average representation of their quality protein, carbohydrate, and dietary fat intake. Distribution of quality protein was determined using the EAA threshold for a meal, which occurs with approximately 10 g. The amount of times the subject met this threshold throughout the day was determined and averaged across the 3-days to give an average representation of their quality protein distribution.

A total body DXA scan was performed using a GE Lunar Prodigy (GE Healthcare, Pewaukee, WI). CAF which includes intra-abdominal fat plus anterior and posterior subcutaneous fat can be distinguished using DXA by identifying the specific region of interest within the analysis program. The region of interest was determined by a rectangle defined at the upper edge of the second lumbar vertebra extended to the lower edge of the fourth lumbar vertebra. The vertical sides of this area were the continuation of the lateral sides of the rib cage [8]. Prior to the DXA scan subjects were asked to refrain from eating for 2-3 hours and were asked to void immediately prior to their test. Females were required to complete an over the counter early pregnancy test prior to participation.

Data were analyzed using Pearson partial coefficient correlations, controlling for body mass and self-reported aerobic and resistance training minutes per week; with an alpha level of 0.05. Data are presented as ± SD. Data from males and females were pooled together to increase statistical power and research indicates that skeletal muscle metabolism of protein does not differ by gender in healthy young humans [9]. Furthermore, Glickman et al. [10] found that this method of estimating CAF provides a valid estimate in both males and females.

## Results

**Figure 1:**
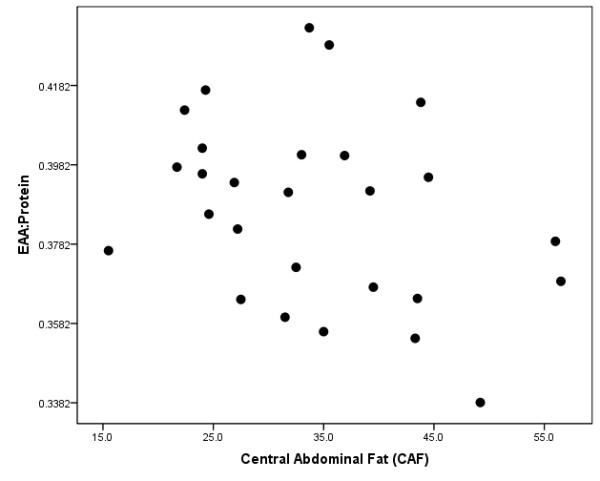
**Scatterplot depicting the relationship between quality protein (Essential Amino Acid:Protein) and percent central abdominal fat (CAF)**.

**Figure 2:**
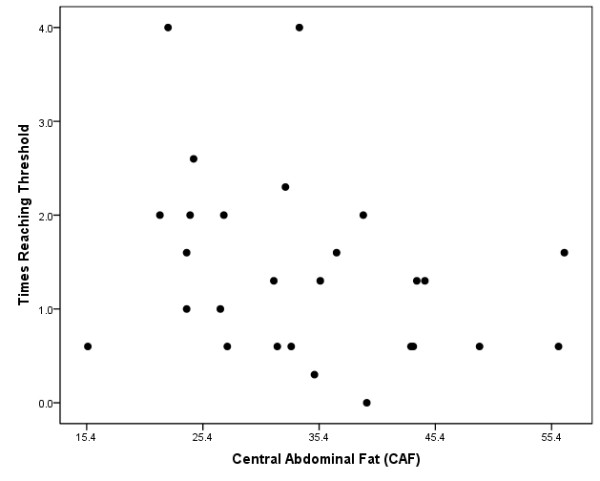
**Scatterplot depicting the relationship between times reaching the essential amino acid threshold and percent central abdominal fat (CAF)**.

Mean values for dietary protein, EAAs, quality protein, and times reaching the EAA threshold have been previously reported (91 g ± 45; 35.9 g ± 19.5; 0.38 ± 0.02; and 1.4 ± 0.9 respectively) [7]. Mean values for carbohydrate and dietary fat intake were 235.3 ± 85.7 g and 72.0 ± 28.6 g respectively. Quality protein consumed in a 24-hour period was inversely related with percent CAF (r = -.420, p = 0.041, Figure 1). No associations were found with carbohydrate (r = -.198, p = 0.354) or dietary fat (r = -.196, p = 0.359) with percent CAF. The amount of times reaching the EAA threshold for a meal throughout the day was also inversely related with percent CAF (r = -.547, p = 0.006, Figure 2).

## Discussion

Currently, the DRI makes no recommendation with regard to the quality of protein consumed, or the distribution of that protein throughout the day. The data from this study demonstrates that both quality and distribution of dietary protein throughout the day is important. The quality and distribution of protein are of particular interest to those who are energy restricted, who might benefit from the consumption of a higher quality protein source (e.g. milk, egg, beef), resulting in a higher EAA content per gram of protein. Neither carbohydrate nor dietary fat intake was associated with percent CAF, which confirms previous data, highlighting the importance of protein intake [6].

Previous research has demonstrated a plateauing of muscle contractile protein synthesis following approximately 9-10 g of EAA; meaning dietary intake of EAAs above this threshold does not significantly contribute to the accretion of skeletal muscle [1]. Researchers have postulated [11] and recently shown that a small difference in the quantity of lean mass has a significant effect on resting energy expenditure [12]. Also, the majority of energy used to provide ATP for muscle protein turnover comes from the oxidation of fat, as this is the preferred energy substrate of muscle at rest [13]. Therefore, a focus on maximizing the muscle synthetic response with ~10 g of EAA may decrease CAF through increased resting energy expenditure from increased lean mass.

In conclusion, moderate to strong correlations between variables indicate that quality and distribution of protein may play an important role in regulating CAF, which is a strong independent marker for disease and mortality. These results warrant further investigation into quality and protein distribution, both of which are currently not covered under the DRI.

## Competing interests

The authors declare that they have no competing interests.

## Authors' contributions

All authors read and approved the final version of the manuscript. JPL, JTB, and TJP participated in the design and coordination of the study. JPL and JTB were jointly responsible for data collection. JPL, JMW, AHM, and MEW were primarily responsible for writing and statistical analysis.
